# Non-Small Cell Lung Carcinoma Cell Motility, Rac Activation and Metastatic Dissemination Are Mediated by Protein Kinase C Epsilon

**DOI:** 10.1371/journal.pone.0031714

**Published:** 2012-02-27

**Authors:** M. Cecilia Caino, Cynthia Lopez-Haber, Joseph L. Kissil, Marcelo G. Kazanietz

**Affiliations:** 1 Department of Pharmacology, Perelman School of Medicine, University of Pennsylvania, Philadelphia, Pennsylvania, United States of America; 2 Molecular and Cellular Oncogenesis Program, The Wistar Institute, Philadelphia, Pennsylvania, United States of America; University of Kentucky College of Medicine, United States of America

## Abstract

**Background:**

Protein kinase C (PKC) ε, a key signaling transducer implicated in mitogenesis, survival, and cancer progression, is overexpressed in human primary non-small cell lung cancer (NSCLC). The role of PKCε in lung cancer metastasis has not yet been established.

**Principal Findings:**

Here we show that RNAi-mediated knockdown of PKCε in H358, H1299, H322, and A549 NSCLC impairs activation of the small GTPase Rac1 in response to phorbol 12-myristate 13-acetate (PMA), serum, or epidermal growth factor (EGF). PKCε depletion markedly impaired the ability of NSCLC cells to form membrane ruffles and migrate. Similar results were observed by pharmacological inhibition of PKCε with εV1-2, a specific PKCε inhibitor. PKCε was also required for invasiveness of NSCLC cells and modulated the secretion of extracellular matrix proteases and protease inhibitors. Finally, we found that PKCε-depleted NSCLC cells fail to disseminate to lungs in a mouse model of metastasis.

**Conclusions:**

Our results implicate PKCε as a key mediator of Rac signaling and motility of lung cancer cells, highlighting its potential as a therapeutic target.

## Introduction

Lung cancer is one of the major causes of cancer-related mortality, with more than 220,000 cases diagnosed and 157,000 deaths estimated for 2010 [1], [2]. The genesis of lung cancer involves the progressive appearance of genetic and epigenetic alterations both in oncogenes and tumor-suppressor genes, ultimately leading to deregulated activation of mitogenic and survival signaling pathways. Common alterations in non-small cell lung cancer (NSCLC), the most prevalent form of lung cancer, include mutations in K-Ras, overexpression of epidermal growth factor receptor (EGFR) and Bcl2, as well as inactivation/down-regulation of p53, Rb, and Pten tumor suppressor genes [3]. Accumulating evidence indicates that protein kinase C (PKC) expression and/or activity is considerably altered in human lung cancer [4], although a causal relationship with disease progression remains to be established. The PKC family consists of three classes of serine-threonine kinases with distinct biochemical and regulatory properties: classical/conventional (cPKCs α, β, and γ), novel (nPKCs δ, ε, η, and θ), and atypical (aPKCs ζ and ι). cPKCs and nPKCs are responsive to phorbol esters and the second messenger diacylglycerol (DAG), a product of PIP2 breakdown by phospholipase C (PLC) in response to activation of tyrosine-kinase and G-protein-coupled receptors. PKC isozymes have paradoxical functional roles depending on the cellular context, ranging from apoptosis to survival, and from mitogenesis to cell cycle inhibition [4], [5].

Despite our extensive knowledge on PKC in cancer development, there is surprisingly limited information regarding the role of individual PKC isozymes in lung cancer progression compared to other epithelial cancers such as skin, breast or prostate cancer, where the involvement of PKCs has been extensively documented [6]. A few available studies established that PKC isozymes display unique functional properties in lung cancer cells. Our laboratory reported that NSCLC cells undergo arrest upon activation of either PKCα or PKCδ. Whereas PKCα induces G2/M arrest and senescence in NSCLC cells, PKCδ induces p21^cip1^ in the G1 phase of the cell cycle [7], [8], [9]. PKCε, on the other hand, drives G1 to S progression of the cell cycle and has a pro-survival role in NSCLC cells, as also described for other cancer cell models [10], [11], [12], [13], [14]. Remarkably, PKCε is abnormally up-regulated in >90% of primary human NSCLCs compared to normal lung epithelium, an effect not observed in small cell lung cancer (SCLC) [10]. It is noteworthy that PKCε has been implicated in oncogenesis in various cancer types. Most notably, ectopic overexpression of PKCε in non-transformed cell lines confers growth advantage or can even lead to malignant transformation [14], [15], [16].

While a large body of evidence links PKC to the promotion of cancer cell invasion and metastatic dissemination, the specific roles of individual PKCs remain only partially understood. [17], [18], [19], [20], [21]. Most notably, phorbol esters promote actin cytoskeleton reorganization and stimulate cell motility in a number of cell lines [22], [23]. Cell type-specific associations between PKC signaling and small Rho GTPases family have been reported. Rac1, a member of the Rho family, has been widely implicated in cytoskeleton rearrangements and cell migration, and play important roles in tumorigenesis and metastasis [24]. PKC has been also implicated in the secretion and expression of basement membrane remodeling proteins, such as matrix metalloproteinases (MMPs) and urokinase-type plasminogen activator receptor (uPAR) [17], [18], [25]. The relationship between individual PKCs and cell motility/invasiveness in lung cancer cells remains to be elucidated.

In this study we focused on PKCε and its potential role in the control of Rac activation and NSCLC cell motility. By means of RNAi and pharmacological approaches we established a key role for PKCε in Rac activation in response to growth factor receptor stimulation, as well as in migration and invasiveness of NSCLC cells. Our studies may have significant implications for the understanding of the molecular mechanisms underlying lung cancer cell metastasis and hopefully underscore novel therapeutic opportunities for treatment of the disease.

## Materials and Methods

### Cell culture

Human NSCLC cell lines (H358, H322, H1299 and A549) were obtained from ATCC and cultured in RPMI 1640 medium supplemented with 10% FBS, 2 mM glutamine, 100 U/ml penicillin, and 100 µg/ml streptomycin. Immortalized human bronchioepithelial cells (HBEC) were a kind gift from Dr. Trevor Penning (University of Pennsylvania) and were cultured on gelatin-coated plates in KSFM medium supplemented with 0.05 mg/ml bovine pituitary extract and 5 ng/ml EGF.

### Adenoviral infections

Cells were infected with adenoviruses (AdVs) encoding β2-chimaerin, dominant-negative Rac (N17-Rac1), PKCα, PKCδ, PKCε or LacZ (control) at the indicated multiplicities of infection (MOIs), as previously reported [9]. Experiments were carried out 24 h later.

### RNAi

Two different RNAi sequences were used for each target. For transient depletion of PKCε we used ON-TARGET Plus RNAi duplexes purchased from Dharmacon (Catalog # J-004653-06, J-004653-08). For EGFR depletion we used ON-TARGET plus SMARTpool (L-003114-00-0005). Silencer Control RNAi #3 (Ambion) was used as control. siRNAs were transfected with LipofectamineRNAi/MAX (Invitrogen). After 48 h cells were serum starved for 24 h and used for the indicated experiments. For stable depletion of PKCε, we used shRNA lentiviral particles from Sigma (Catalog #SHCLNV-NM_005400, clone ID x-741s1c1 and x-375s1c1). Upon infection, cells were selected for 2 weeks with puromycin (1–2 µg/ml depending on the cell line), as previously described [26].

### Rac-GTP pull-down assays

Determination of Rac-GTP levels was carried out essentially as previously described [27]. Briefly, cells growing at low confluency were serum starved for 24 h and stimulated with PMA, FBS or EGF at the concentrations indicated. Cells were lysed in pull-down buffer (20 mM Tris-ClH pH 7.4, 150 mM NaCl, 5 mM MgCl2, 0.5% NP40, 5 mM β-glycerophosphate, 1 mM DTT and protease inhibitors) containing 15 µg/ml of PBD-GST. Lysates were cleared by centrifugation (10 min at 4°C, 13,000×*g*) and incubated with glutathione-Sepharose beads (GE Healthcare) for 45 min at 4°C. After centrifugation, Rac-GTP bound to the beads was washed twice with pull-down buffer and run on SDS-PAGE. Rac1 was detected by Western blot using an anti-Rac1 antibody (Upstate Biotechnology, 1∶1000 dilution).

### Cytoskeleton morphology assays

Cytoskeleton morphology was assessed as described previously [26]. Briefly, cells growing on glass coverslides at low confluency were serum starved for 24 h and stimulated with PMA or FBS at the concentrations indicated. Following fixation with 4% formaldehyde, F-actin was stained with phalloidin-rhodamine and nuclei were counter-stained with DAPI. Slides were visualized by fluorescence microscopy and 5 random fields were scored for presence/absence of ruffles.

### Wound assays

Experiments were carried out in triplicate plates essentially as previously described [26].Three micrographs were obtained at time = 0 h and 9–24 h later depending on the cell line. Wound closure measurements were normalized to the maximum initial scratch area for each well.

### Migration in Boyden chambers

Cells were serum starved for 24 h, trypsinized, suspended in 0.1% BSA/RPMI, and seeded (1.5×10^4^ cells/well) in the upper compartments of the chemotaxis chamber (NeuroProbe). Polycarbonate membranes of 8 µM pore diameter were used to separate the upper and lower compartments. In the lower chamber, RPMI medium containing 10% FBS was used. After an incubation of 18 h at 37°C, membranes were recovered and cells on the upper side (non-migratory) wiped off the surface. Cells on the lower side of the membrane were fixed and stained with DIFF Quik Stain Set (Dade Behring). Migratory cells in each well were counted by contrast microscopy in 5 independent fields.

### Invasion assays and determination of proteases

Invasion assays using Matrigel-coated polycarbonate membranes were conducted as previously described [5]. The mRNA levels of extracellular matrix (ECM) proteases and protease inhibitors were determined by qPCR as described previously [28]. Briefly, RNA was isolated with the QIAGEN RNeasy kit, reverse transcribed, and assayed for simultaneous detection of ECM-related genes and 2 housekeeping genes by qPCR using RT2 Profiler PCR Array plates (QIAGEN) and a RT2 SYBR Green/5-carboxy-X-rhodamine (ROX) qPCR master mix. Data were normalized using GAPDH and β-actin as housekeeping genes, and the relative levels of mRNA calculated according to the ΔCt method. Genes that displayed >1.5-fold change in expression as a consequence of PKCε depletion were selected for further analysis.

For the determination of proteases in conditioned medium (CM), cells (3×10^6^) were incubated for 48 h in serum-free medium and the supernatant concentrated using an Amicon Ultra-4 filtration device with a 30 kDa cut-off (Millipore). The concentrated CM was used for detection of proteases with a RayBiotech antibody array following the manufacturer's instructions, or for gelatin zymography, as described previously [16].

### Cell adhesion

Plates were coated with 500 µg/ml Matrigel, blocked with 0.5% BSA and cells (3×10^4^ cells/well in 0.1% BSA/RPMI) were seeded in triplicate. FBS (10%) was added to stimulate adhesion and after incubation for 18 h at 37°C unattached cells were washed away with PBS, fixed in methanol and stained with crystal violet solution (3.5 mg/ml crystal violet, 19% ethanol, 1% methanol). After solubilization in 2% SDS, optical density was measured at 570 nm.

### Experimental metastasis assays and survival in nude mice

Studies were carried out in strict accordance to the recommendations in the Guide for the Care and Use of Laboratory Animals of the National Institutes of Health. Protocols were approved by the Institutional Animal Care and Use Committee (IACUC) of the University of Pennsylvania (Protocol number 802268) and The Wistar Institute (Protocol number 112318). Cells were grown to 80% confluency and suspended in PBS. Male athymic mice (6–8 weeks, Harlan Laboratories) were injected *i.v.* with 5×10^5^ cells in 0.1 ml (10 mice/group). After 100 days, mice were sacrificed and the lungs removed, fixed in 4% formaldehyde/PBS, paraffin embedded, dissected and stained with hematoxilin/eosin (H&E). The incidence of metastasis was determined as described [29], [30]. To determine the area of metastatic lesions, digital images of 2 sections from the 5 lobules from each lung were processed with ImagePro 6.2, as described previously [31]. For survival studies, nude mice (10 mice/group) were injected with 2×10^6^ cells and the time of death of each animal was recorded.

### Western blots

Western blots and densitometric analyses were carried out as previously described [9], [27]. Bands were visualized by Enhanced Chemiluminescence (ECL) Western blotting detection system. Images were captured using a FUJIFILM LAS-3000 system and the LAS-2000 software.

### Statistical analysis

Averages of at least 3 independent experiments were compared with a *t*-test using GraphPad Prism 3.0. For metastasis experiments, the distribution of each group was compared against the distribution of the control, using a Mann-Withney non-parametric test. In all cases, a p<0.05 was considered statistically significant.

## Results

### PKCε mediates PMA-induced Rac activation and Rac-mediated responses

**Figure 1:**
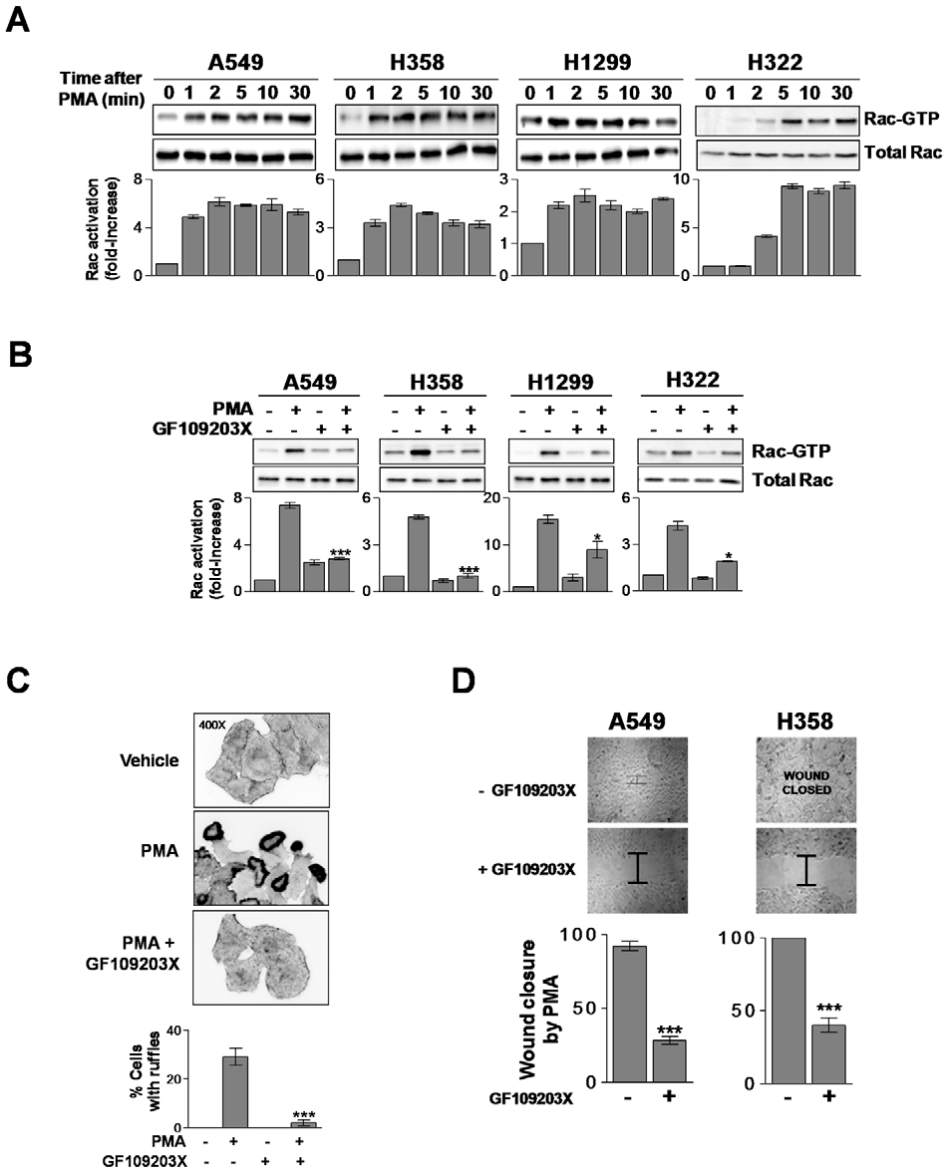
PMA activates Rac responses in NSCLC cells. A. NSCLC cells were serum starved for 24 h and treated with PMA (100 nM) for different times, and Rac-GTP assays determined using a pull-down assay. A representative experiment is shown. The fold-induction in Rac-GTP levels normalized to total Rac at time = 0, as determined by densitometry, is represented in the bottom graphs. Data are expressed as mean ± S.D. (n = 3). B. Effect of the pan-PKC inhibitor GF109203X (5 µM, 30 min) on Rac activation by PMA (100 nM, 2 min).The fold-induction in Rac-GTP levels normalized to total Rac at time = 0, as determined by densitometry, is represented in the bottom graphs. Data are expressed as mean ± S.D. (n = 3). *, p<0.05; ***, p<0.001. C. A549 cells were treated with PMA (100 nM, 30 min), fixed, and stained with phalloidin-rhodamine. *Top*, representative micrographs are shown (n = 3). *Bottom*, quantification of cells bearing ruffles, expressed as mean ± S.D. of 3 individual experiments. ***, p<0.001. D.Confluent monolayers of cells were treated with PMA (100 nM, 30 min) and scraped. Closure of wounds was recorded at 9 h (A549 cells) or 20 h (H358 cells) post-treatment. Experiments were carried out in triplicate plates. Results are expressed as percentage of the closure induced by PMA, and expressed as mean ± S.E.M. (n = 3). ***, p<0.001.

To determine if PKCs play a role in the regulation of Rac activity in NSCLC cells, we first examined the effect of phorbol 12-myristate 13-acetate (PMA) on Rac activation in four different NSCLC cell lines (A549, H358, H1299, and H322). Cells were treated with 100 nM PMA for different times (0–30 min) and the levels of Rac1-GTP (active Rac) assessed using a PBD pull-down assay. As shown in Fig. 1A, PMA induced a fast activation of Rac1 in all four NSCLC cell lines that begins at 1–2 min and is sustained for at least 30 min. As PMA can directly activate targets unrelated to PKC that modulate the activity of small GTPases (such as chimaerins, MRCK or RasGRPs), we examined the effect of the pan-PKC inhibitor GF19203X (bisindolmaleimide I) [32]. Rac1 activation by PMA was indeed sensitive to the pan-PKC inhibitor GF109203X, particularly in A549, H358, and H322 cells, suggesting that the effect of the phorbol ester is mediated primarily through PKC activation (Fig. 1B).

Rac mediates the reorganization of the actin cytoskeleton into ruffles and lamellipodia, a critical step for cell motility [33], [34]. When A549 cells were treated with PMA, a marked induction of circular ruffles was observed. This effect was essentially abolished by GF109203X (Fig. 1C). Similar results were observed in H358, H1299 and H322 cells (Fig. S1). To determine if PMA activates NSCLC cell motility we used a wound assay. In comparison to vehicle treatment, which closed the wound by 50–70% depending on the NSCLC cell line, full closure of the wound was observed in response to PMA (Fig. S2). PMA-induced NSCLC cell motility was markedly reduced by the PKC inhibitor GF109203X (Fig. 1D and Fig S2). These results argue for the involvement of phorbol ester/DAG responsive PKCs, namely cPKCs and/or nPKCs, in Rac activation in NSCLC cells.

**Figure 2:**
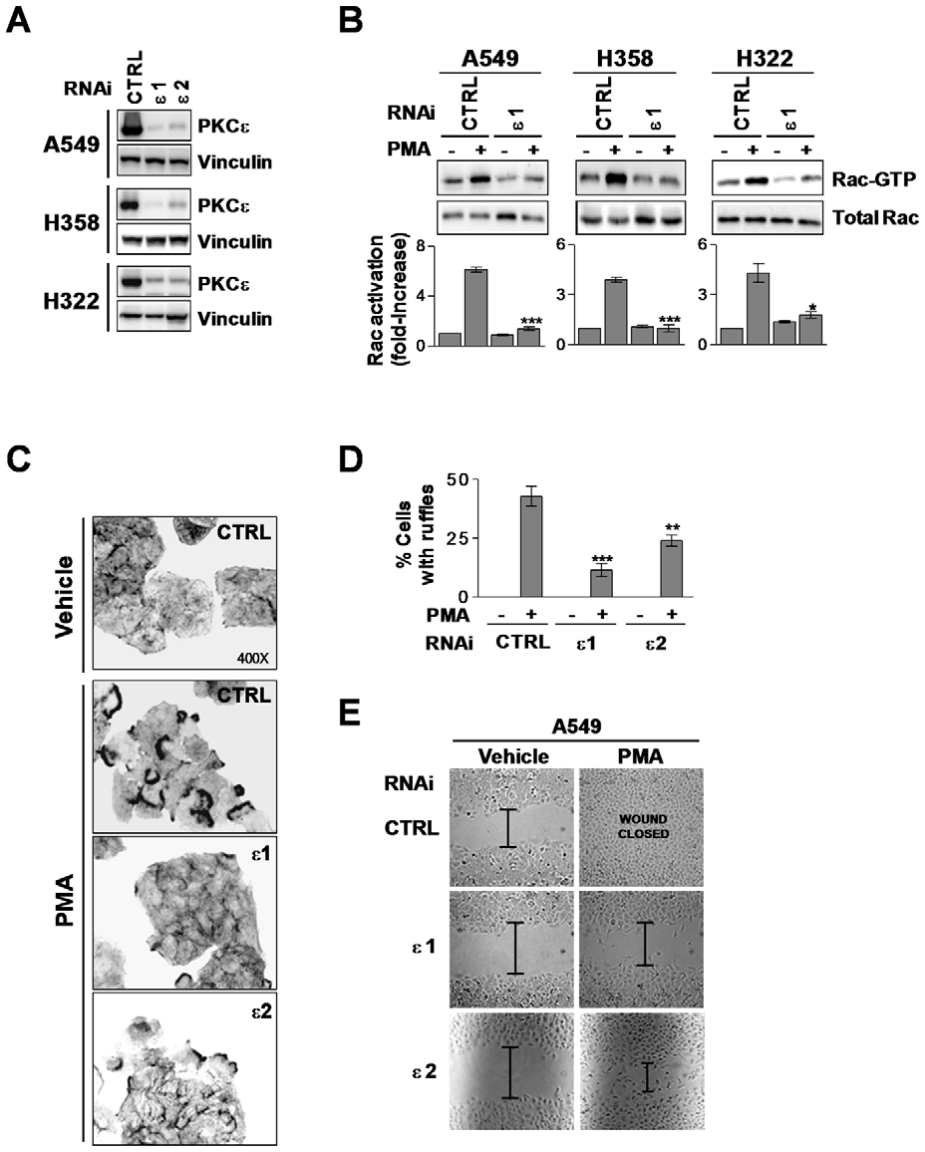
PKCε mediates Rac activation and Rac-mediated responses induced by PMA. A. NSCLC cells were transfected with either PKCε RNAi (ε1 or ε2) or control (*CTRL*) RNAi and serum starved for 24 h. A representative Western blot for PKCε expression at the time of PMA treatment is shown (n = 3). B. Cells subject to PKCε or control RNAi were stimulated with PMA (100 nM, 2 min) and Rac-GTP levels determined using a pull-down assay.A representative experiment is shown. The fold-induction in Rac-GTP levels normalized to total Rac at time = 0, as determined by densitometry, is represented in the bottom graphs. Data are expressed as mean ± S.D. (n = 3). *, p<0.05; ***, p<0.001. C. A549 cells were treated with PMA (100 nM, 30 min), fixed, and stained with phalloidin-rhodamine. Representative micrographs are shown (n = 3). D. Quantification of A549 cells bearing ruffles, expressed as mean ± S.D. of 3 individual experiments. **, p<0.01; ***, p<0.001. E. Closure of wounds in response to PMA (100 nM, 30 min) was recorded at 9 h (A549 cells). Experiments were carried out in triplicate plates. Results are expressed as percentage of the closure induced by PMA, and expressed as mean ± S.E.M. (n = 3). ***, p<0.001.

NSCLC cells express very high levels of PKCε compared to normal lung epithelial cells (Fig. S3), as also observed in human NSCLC specimens [10]. To determine if PKCε is implicated in the regulation of Rac activation and cell motility in NSCLC cells we knocked-down PKCε using RNAi. Two different siRNA duplexes (ε1 and ε2) were used, which led to >80% depletion in all NSCLC cell lines used (Fig. 2A). Notably, activation of Rac1 by PMA in PKCε-depleted NSCLC cells was markedly reduced relative to their corresponding control cells transfected with a non-target RNAi sequence (Fig. 2B). Moreover, PKCε knock down in A549 reduced phorbol ester-induced ruffle formation (Fig. 2C and 2D) and wound closure (Fig. 2E). Similar results were observed in H322 cells (Fig. S4). Our results clearly establish the involvement of PKCε in PMA-induced Rac activation and motility in NSCLC cells.

### PKCε is required for growth factor-induced Rac activation and NSCLC cell motility

**Figure 3:**
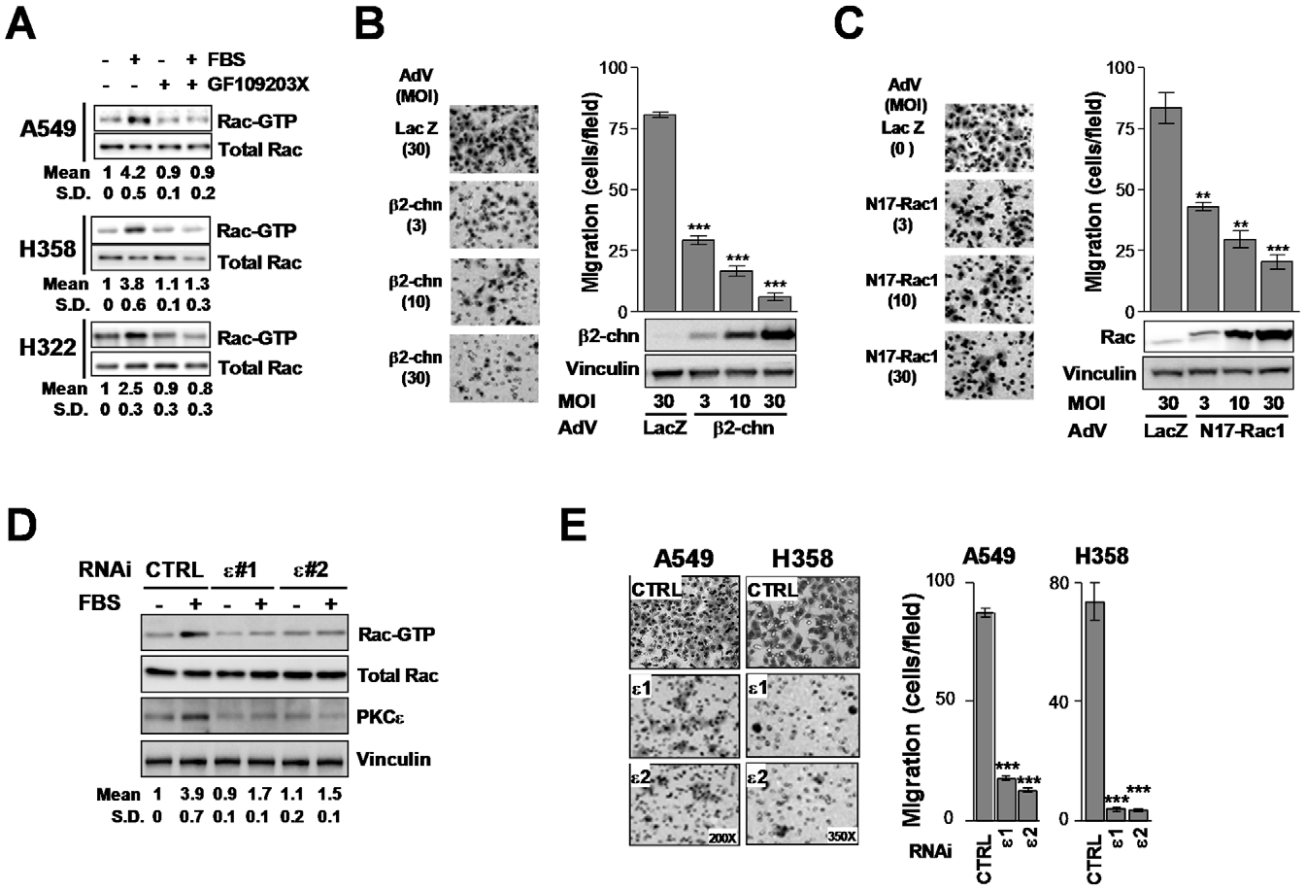
Rac responses induced by serum are mediated by PKCε. A. NSCLC cells were serum starved for 24 h and then incubated with 10% FBS (5 min). Rac-GTP levels determined using a pull-down assay. A representative experiment is shown and the induction of Rac, determined by densitometry, is provided at the bottom of Western blot. Data are expressed as mean ± S.D. (n = 3). B and C. A549 cells were infected with AdVs for either β2-chimaerin (*β2-chn*) (*Panel C*) or N17-Rac1 (*Panel D*) at the MOIs indicated in the figure. A LacZ AdV was used as control. Migration in response to FBS was assayed using a Boyden chamber for 16 h. *Left panels*, representative micrographs. *Right panels*, quantification of 3 independent experiments. Data are expressed mean ± S.E.M. (n = 3). **, p<0.01; ***, p<0.001. D. Rac activation by 10% FBS (2 min) in A549 cells subject to either PKCε or control RNAi, as determined using a pull-down assay. A representative experiment is shown and the induction of Rac, determined by densitometry, is provided at the bottom of each Western blot. Data are expressed as mean ± S.D. (n = 3). E. Migration of A549 and H358 cellssubject to either PKCε or control RNAi in response to 10% FBS (16 h) using a Boyden chamber. *Left panels*, representative micrographs. *Right panels*, quantitation of 3 independent experiments. Data are expressed mean ± S.E.M. (n = 3). **, p<0.01; ***, p<0.001.

To assess the involvement of PKCs in Rac activation in NSCLC cells in response to physiological stimuli, we first examined the effect of the PKC inhibitor GF109203X on serum responses. A549, H358 and H322 cells were serum-starved for 24 h and Rac-GTP levels determined in response to acute stimulation with serum. Addition of FBS activated Rac1 in all NSCLC cell lines, and this effect was essentially abolished by GF109203X (Fig. 3A). The requirement of Rac for NSCLC cell migration was establishedby means of expressing either the RacGTPase activating protein (Rac-GAP) β2-chimaerin [35] or a dominant-negative Rac mutant (N17-Rac1) using adenoviral means. Either β2-chimaerin or N17-Rac1 impaired NSCLC cell motility. The effect was proportional to the expression levels achieved by increasing the multiplicities of infection (MOI) of the corresponding AdVs (Fig. 3Band 3C).

Next, we wished to examine if PKCε mediates the activation of Rac1 by serum. As shown in Fig. 3D, depletion of PKCε with either ε1 or ε2 RNAi duplexes impaired Rac1 activation induced by FBS in A549 cells. In addition, NSCLC cell migration induced by serum was essentially abolished when PKCε was knocked-down (Fig. 3E) without affecting cell viability (Fig. S5). GF109203X also inhibited cell migration, although prolonged incubation with the “pan” PKC inhibitor, as required in the motility assay, reduced cell viability (Fig. S5). The involvement of PKCε in NSCLC cell motility was further confirmed by means of an overexpression approach. Adenoviral delivery of PKCε to H358 or A549 cells enhanced cell motility. This effect was not observed upon overexpression of PKCα or PKCδ (Fig. S6). These results clearly implicate PKCε as a mediator of Rac responses in NSCLC cells.

### EGF signals to Rac via a PLCγ-PKCε-dependent pathway

**Figure 4:**
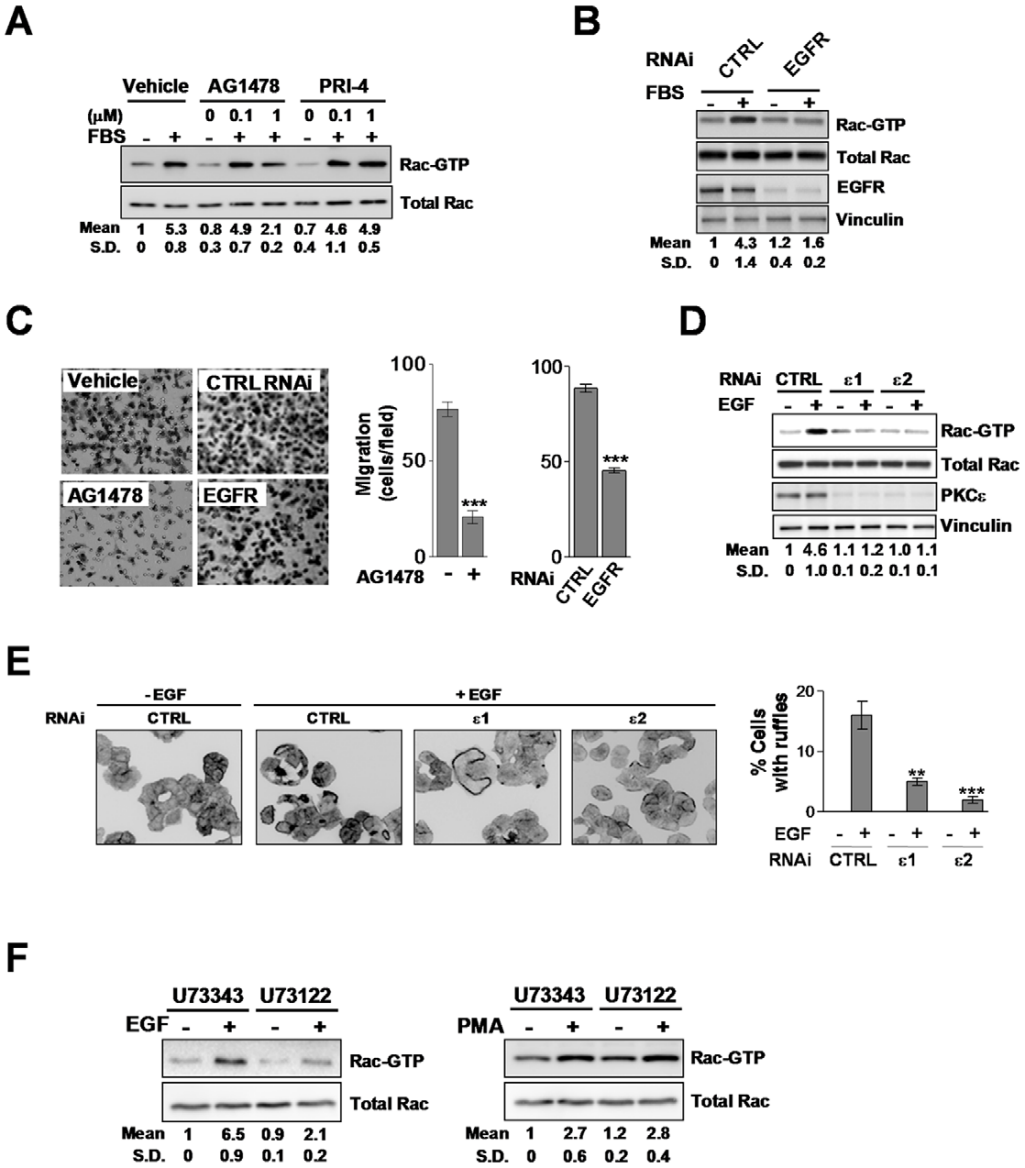
EGF-induced activation of Rac and Rac-mediated responses in A549 cells is mediated by PLC and PKCε. A. Effect of AG1478 (0.1–1 µM) and PRI-4 (0.1–1 µM) on the activation of Rac by 10% FBS (2 min). A representative experiment is shown. Values indicate the fold-induction in Rac-GTP levels normalized to total Rac relative to levels at time = 0, as determined by densitometry. Data are expressed as mean ± S.D. (n = 3). B. Effect of EGFR depletion on the activation of Rac by 10% FBS (2 min).Values indicate the fold-induction in Rac-GTP levels normalized to total Rac at time = 0, as determined by densitometry. Data are expressed as mean ± S.D. (n = 3). C. Effect of AG1478 (1 µM) or EGFR RNAi on 10% FBS-induced A549 cell migration using a Boyden chamber (16 h). *Left panels*, representative micrographs. *Right panel*, quantification of 3 independent experiments. Data are expressed mean ± S.E.M. (n = 3). ***, p<0.001. D.A549 cells subject to either PKCε or control (*CTRL*) RNAi were serum starved for 24 h and stimulated with EGF (100 ng/ml, 2 min). Rac activation was determined with a pull-down assay. Data are expressed as mean ± S.D. (n = 3). E. Induction of ruffle formation by EGF (100 ng/ml, 15 min) in A549 cells subject to either PKCε or control (*CTRL*) RNAi. *Left*, representative micrographs are shown (n = 3). *Right*, quantification of cells bearing ruffles, expressed as mean ± S.D. of 3 individual experiments. ***, p<0.001. F. Effect of U73343 or U73122 (10 µM, 30 min) on Rac activation by either EGF (100 ng/ml) or PMA (100 nM, 2 min) in A549 cells. Values indicate the fold-induction in Rac-GTP levels normalized to total Rac at time = 0, as determined by densitometry. Data are expressed as mean ± S.D. (n = 3).

EGF and PDGF are potent activators of Rac, and they induce the formation of circular ruffles in a similar manner than PMA and FBS (see Fig. 1D and also [36], [37]). To determine if these growth factors could be mediating NSCLC cell motility induced by FBS, we used EGFR or PDGFR pharmacological inhibitors. Rac pull-down assays revealed that the EGFR inhibitor AG1478 greatly reduced FBS-induced Rac1 activation in A549 cells. On the other hand, the PDGFR inhibitor PRI-4 was ineffective (Fig. 4A). To confirm the involvement of EGFR we knocked it down using RNAi duplexes. We found that Rac1 activation in response to FBS was markedly inhibited in EGFR-depleted A549 cells (Fig. 4B), thus reinforcing the conclusions obtained with the EGFR inhibitor. Both AG1478 and EGFR RNAi also inhibited FBS-induced A549 cell motility (Fig. 4C). Furthermore, treatment of A549 cells with EGF (100 ng/ml, 2 min) caused a ∼5-fold induction in Rac-GTP levels, and this effect was essentially abolished upon PKCε depletion (Fig. 4D). Likewise, ruffle formation in response to EGF was inhibited as a consequence of PKCε depletion (Fig. 4E).

PKCε is a downstream effector of EGFR, and it translocates to the plasma membrane in response to EGF in a DAG-dependent manner ([38], and data not shown). As EGFR couples to the DAG-generating enzyme PLCγ in response to stimulation [39], we reasoned that inhibition of PLC should affect Rac activation by EGF. A549 cells were treated with the PLC inhibitor U73122 or its inactive analogue U73343 and stimulated with EGF. U73122 treatment prevented Rac1 activation by EGF, whereas its inactive analogue did not. As an additional control we used PMA, which activates Rac1 regardless of PLC inhibition (Fig. 4F). Altogether, these results argue for the requirement of a PLCγ-dependent activation of PKCε for the activation of Rac1 by EGF. Thus, PKCε is a downstream effector of EGFR in NSCLC cells.

### Pharmacological inhibition of PKCε affects NSCLC cell migration

**Figure 5.The:**
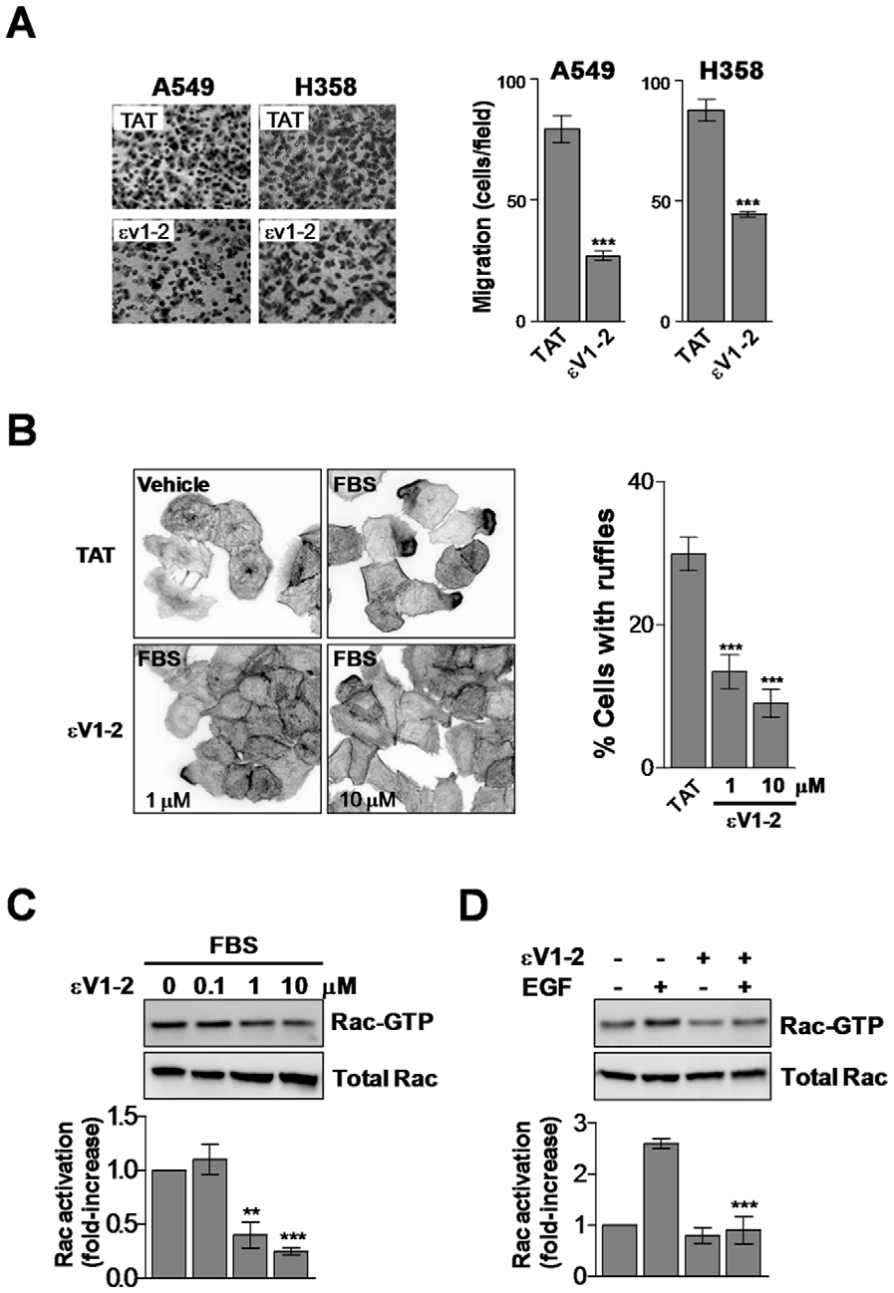
PKCε inhibitor εV1-2 impairs the formation of ruffles and migration in NSCLC cells. A. A549 or H358 cells were serum starved for 24 h and migration in response to 10% FBS for 16 h determined using a Boyden chamber in the presence of either εV1-2 or control TAT (1 µM). *Left panels*, representative micrographs. *Right panels*, quantification of migratory cells. Data are expressed as the mean ± S.D. of 3 individual experiments. ***, p<0.001. B. Induction of ruffle formation by 10% FBS (30 min) in A549 cells in the presence of increasing concentrations of εV1-2. *Left*, representative micrographs. *Right*, quantification of cells bearing ruffles expressed as mean ± S.D. of 3 individual experiments.***, p<0.001. C. Effect of εV1-2 on Rac activation by 2% FBS (2 min) (*left panel*) or EGF (10 ng/ml, 2 min) (*right panel*). The concentration of εV1-2 in the EGF experiments was 1 µM. Representative experiments are shown. Values indicate the fold-induction in Rac-GTP levels normalized to total Rac relative to levels at time = 0, as determined by densitometry. Data are expressed as mean ± S.D. (n = 3).**, p<0.01; ***, p<0.001.

To further establish the requirement of PKCε for Rac1 activation in NSCLC cells we used εV1-2, a specific inhibitor of PKCε translocation that has been widely used in cellular and animal models [40], [41], [42], [43]. A549 and H358 cells were incubated with either εV1-2 or the control peptide (TAT) and analyzed for changes in motility. In agreement with results observed using PKCε RNAi, pharmacological inhibition of PKCε significantly reduced the migratory ability of NSCLC cells in response to FBS (Fig. 5A and Fig. S7) without affecting cell viability (Fig. S5). εV1-2 also inhibited ruffle formation in response to FBS (Fig. 5B). Finally, elevation of Rac-GTP levels in response to FBS or EGF was diminished by pharmacological inhibition of PKCε (Fig. 5C).

### PKCε is implicated in invasiveness and metastasis of NSCLC cells

**Figure 6:**
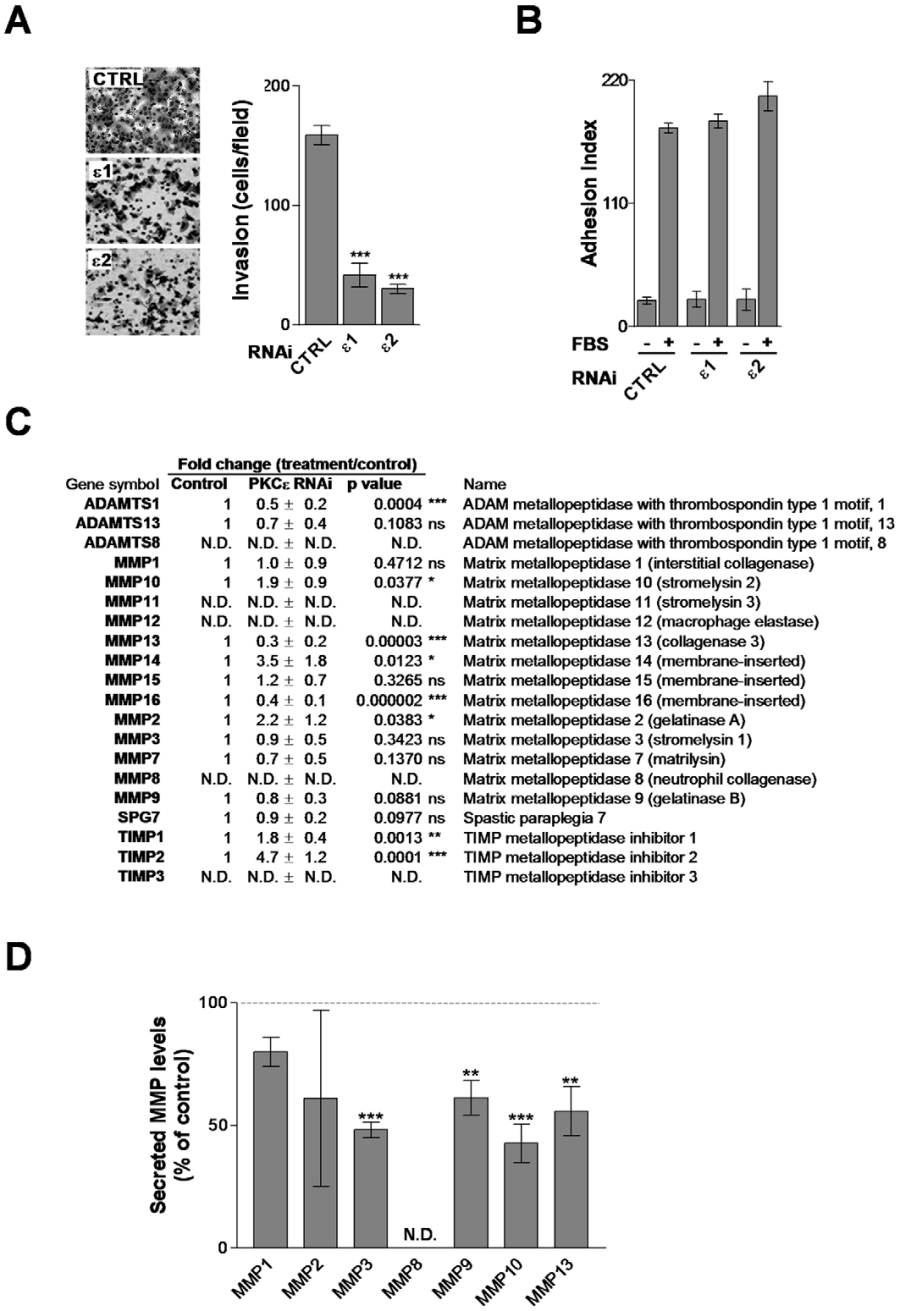
PKCε modulates invasion and ECM-genes in A549 cells. A. A549 cells were transfected with RNAi for either PKCε (ε1 and ε2) or control (*CTRL*) and 2 days later serum-starved for additional 24 h. Cells were then seeded in Boyden chambers with Matrigel-coated membranes. Invasion was quantified 24 h later. *Left*, representative micrographs. *Right*, quantification of invasive cells. Data are represented as mean ± S.E.M. (n = 3). ***, p<0.001. B. Adhesion to Matrigel-coated plates was determined 24 h after seeding. Data are represented as mean ± S.E.M. (n = 3). C. Analysis of proteases and inhibitors of proteases by qPCR in control or PKCε-depleted cells. Results are expressed as mean ± S.E.M. (n = 4). D. Detection of ECM proteases secreted to conditioned medium (CM) from control or PKCε-depleted A549 cells. Quantification of MMP levels by densitometry is represented as mean ± S.E.M. (n = 3). **, p<0.01; ***, p<0.001. *N.D.*, not-determined.

The ability of cancer cells to invade and metastasize is associated with the production and release of proteases that remodel the extracellular matrix (ECM) as well as changes in cell adhesion [44]. To determine if PKCε is involved in NSCLC cell invasiveness we assessed migration through Matrigel using a Boyden chamber. Fig. 6A shows that PKCε-depleted A549 cells essentially lost their ability to migrate through Matrigel (Fig. 6A). On the other hand, knocking down PKCε did not affect the adhesion of A549 cells to Matrigel (Fig. 6B). These findings suggest that PKCε plays a critical role in the remodeling of the matrix and prompted us to determine the expression of relevant ECM proteases and protease inhibitors. As a first approach we used a commercial qPCR array. Interestingly, mRNA levels of ADAMTS1, MMP13, and MMP16 metalloproteinases were down-regulated in PKCε-depleted A549 cells. PKCε depletion also caused a marked induction in mRNA levels of overall the protease inhibitors TIMP1 and TIMP2, suggesting an imbalance in protease activity (Fig. 6C). As a complementary approach, we also determined levels of metalloproteinases secreted to the medium using an Antibody Array. Secretion of MMP3, MMP9, MMP10, and MMP13 from PKCε-depleted cells to conditioned medium was markedly reduced compared to control (Fig. 6D). The reduction in MMP9 activity by PKCε depletion was confirmed by zymography (Fig. S8). Thus, PKCε may affect the synthesis and/or release of specific proteases from NSCLC cells.

**Figure 7:**
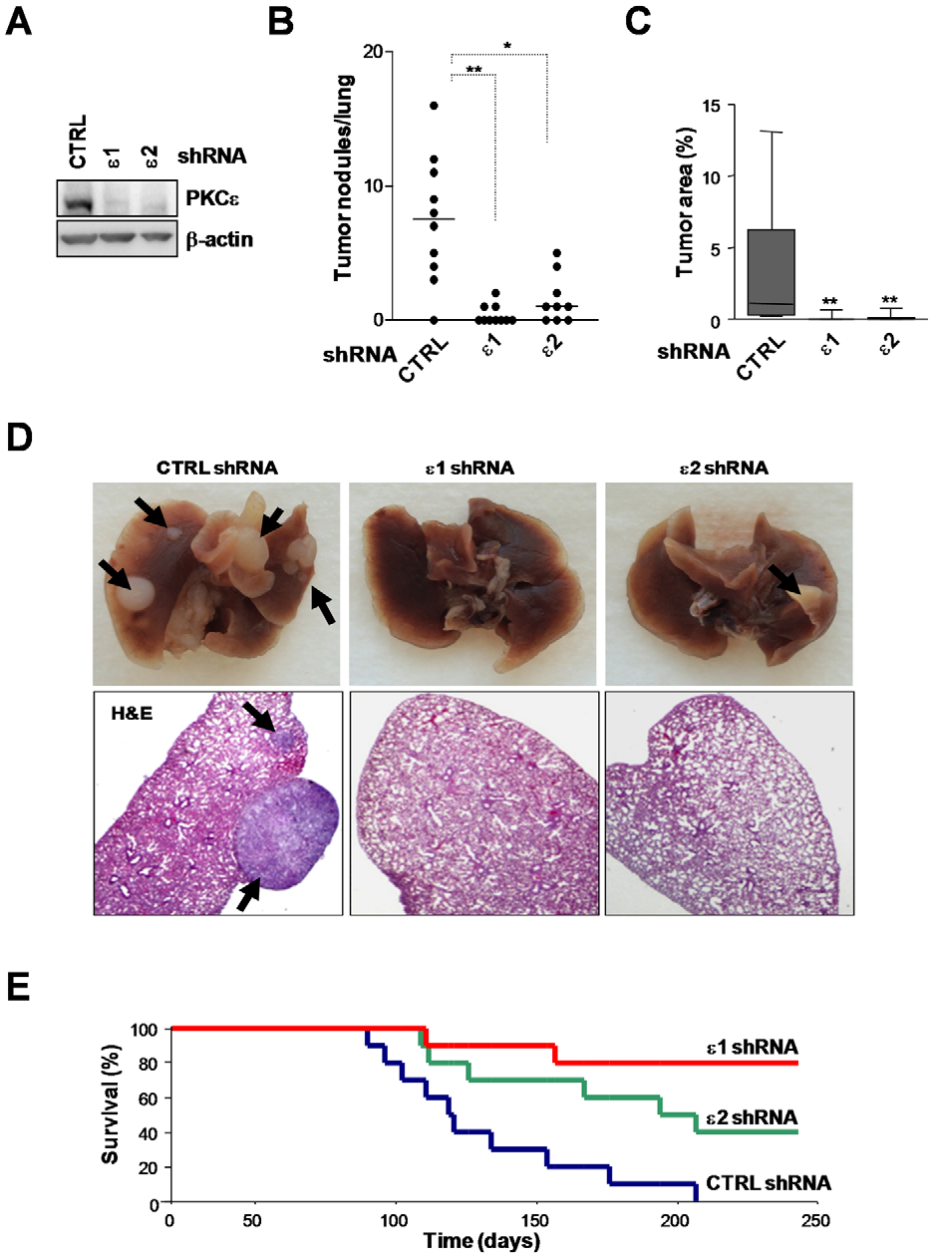
PKCε plays a role in invasion and metastatic dissemination of NSCLC cells. A549 cells were subject to stable PKCε depletion using two different shRNA lentiviruses (ε1 and ε2) or control cells (*CTRL*, non-target shRNA lentivirus) and selected with puromycin. Cells were inoculated *i.v.* into athymic mice. A. Levels of PKCε depletion achieved in stable cells lines. A representative Western blot is shown. B. Incidence of metastasis per lung in mice sacrificed 100 days post-inoculation of cells. *, p<0.05; **,p<0.01. C. Area of tumors as determined in microscopic images. **, p<0.01. D. Representative images of lungs from mice inoculated with PKCε-depleted or control cells. *Arrows*, metastatic foci in whole-fixed lungs (*top panels*) or in lungs stained with hematoxilin and eosin (H&E) (*bottom panels*). E. Overall survival of mice inoculated with PKCε-depleted or control A549 cells.

To establish the relevance of our findings in a metastasis model *in vivo*, we examined the ability of A549 cells to form lung tumors after *i.v.* inoculation into athymic nude mice. For these experiments we generated stable PKCε-depleted A549 cell lines (puromycin-selected) using two different shRNA lentiviruses. A control cell line was generated by infection with a non-targeting shRNA lentivirus (Fig. 7A). Mice were sacrificed 100 days after inoculation. Notably, whereas control cells readily form macroscopic lung tumors in nude mice (7.5±1.5 tumor nodules/lung), PKCε-depleted cells failed to form tumor nodules (0.4±0.2 and 1.5±0.5 tumor nodules/lung for ε1 and ε2, respectively) (Fig. 7B). Quantification of the tumor areas in histological sections of the lungs also showed remarkable differences between control and PKCε-depleted A549 cells (Fig. 7C). Representative pictures of lung tumors and H&E staining are depicted in Fig. 7D.

Finally, we carried out a survival experiment in athymic nude mice. A549 cells subject to stable PKCε-depletion or control cells were injected into the tail vein of athymic mice, and survival of the animals was followed for >8 months. As shown in Fig. 7E, whereas 100% of mice injected with control A549 cells died at ∼200 days after inoculation, those injected with PKCε-depleted cells have overall survival rates of 80% (PKCε shRNA #1) and 50% (PKCε shRNA #2) at 250 days. Altogether, these experiments suggest an important role for PKCε in NSCLC cell invasion and metastatic dissemination.

## Discussion

Although the involvement of PKCs in carcinogenesis has been recognized for decades, the specific roles of individual PKC isoforms during specific stages of cancer progression remain only partially understood, particularly in metastatic dissemination. This is evident in lung cancer, where PKCs, mainly those isoforms responsive to DAG/phorbol esters (cPKCs and nPKCs), have been poorly studied. Here we demonstrate that PKCε plays an essential role in lung cancer cell motility and invasiveness. Our studies identified PKCε as a key regulator of Rac1 activation in response to stimuli in NSCLC cells. Notably, RNAi depletion or pharmacological inhibition of PKCε impairs Rac1 activation as well as Rac-dependent responses, including ruffle formation, migration, and invasion. Rac1, a member of the Rho family of small GTPases plays crucial roles in the reorganization of actin cytoskeleton and motility, and inhibition of Rac1 or its downstream effectors severely impairs cell migration and dissemination of cancer cells [45]. Studies have shown that Rac is required for migration of NSCLC cells [46], which we validated in this study using a dominant-negative Rac mutant and by expressing the Rac-GAP β2-chimaerin. Aberrant expression of Rac exchange factors (Rac-GEFs) Ect2 and Vav1/2/3 has been reported in lung cancer [47], [48], [49], suggestive of a crucial role for the Rac signaling pathway in the progression of the disease.

Emerging evidence in the last years strongly argues for a connection between PKC isozymes and the invasive phenotype. Several reports have directly or indirectly suggested a link between PKCε and invasiveness, and PKCε was found to be highly overexpressed in lung cancer as well as in other epithelial cancers [14], prompting us to reason that this PKC may be highly relevant for NSCLC metastatic dissemination. Indeed, early studies showed that NIH 3T3 fibroblasts overexpressing PKCε acquire a polarized morphology and form invadopodial-like structures [20], and that PKCε signals downstream of β1-integrin to modulate cell spreading [14]. PKCε regulates motility and invasion in models of breast as well as head and neck cancer, at least partially through RhoA and RhoC GTPases [19], [50]. Moreover, skin transgenic overexpression of PKCε in mice leads to the development of metastatic squamous carcinomas [51]. A link between PKCε and Rac has been reported in lung endothelial cells downstream of the sphingosine-1-phosphate receptor [52]. Experiments presented in our study show that there are major changes in the expression of proteases related to invasiveness upon depletion of PKCε from NSCLC cells. A number of MMPs (ADAMTS1, MMP1, MMP2, MMP10, MMP13, MMP14 and MMP16) are down-regulated at the mRNA level, whereas the protease inhibitors TIMP1 and TIMP2 are up-regulated in PKCε knockdown NSCLC cells. Furthermore, the overall secretion of several MMPs from NSCLC cells was markedly reduced by PKCε RNAi. Although the requirement for Rac in the regulation of protease/protease inhibitors in NSCLC cells remains to be determined, recent studies in different cellular models identified Rac signaling as a requirement for the secretion of MMP9, MMP2 and MMP1 [53], [54], [55]. Moreover, in lung cancer cells Rac mediates PKCι-induced secretion of MMP10 [56], suggesting common downstream targets for PKC isozymes controlling invasion. Thus, it is conceivable that PKCε overexpression signals via Rac to impact on the expression of multiple proteases, leading ultimately to enhanced invasiveness of lung cancer cells.

Our results identified PKCε as an essential mediator of Rac1 activation downstream of the EGFR, a receptor that couples to PLCγ to generate DAG in response to its activation [39]. In PKCε-depleted NSCLC cells EGF fails to activate Rac or Rac-dependent responses, including ruffle formation and motility. Pharmacological inhibition of PLC impairs Rac activation by EGF, arguing that DAG-mediated recruitment of PKCε to the plasma membrane plays a crucial role in the activation of the Rac signaling pathway. How PKCε controls Rac activation downstream of EGFR (or eventually other tyrosine-kinase receptors) remains unexplored, hence identifying such mechanisms merits further investigation. One attractive mechanism may involve the activation of exchange factors for Rac (Rac-GEFs) through phosphorylation. There are at least 25 Rac-GEFs in the genome, and their relative expression in lung cancer has not been fully established. Using a Rac-GEF qPCR array we recently found that NSCLC cell lines express high levels of Vav1/2, Ect2, DEPC2 and DEPC1 compared to normal cells (M.C.C. and M.G.K., unpublished studies). Elegant studies by Fields and coworkers demonstrated that Ect2 is required for the oncogenic activity of PLCι, a member of the atypical PKC family. Ect2 is overexpressed in lung cancer and is an essential component of the PKCι-Par6 complex that controls cell polarity. RNAi-mediated knockdown of Ect2 in A549 cells inhibits Rac1 activity as well as it impairs growth, tumorigenicity and invasion of NSCLC cells [48]. However, upon RNAi depletion of Ect2 from A549 cells we could not find any significant changes in Rac1 activation induced by EGF (C.L.H. and M.G.K., unpublished studies). These results argue for major differences in the regulation of Rac signaling by DAG-responsive and DAG-unresponsive PKCs. Vav isoforms, Rho/Rac-GEF overexpressed in nearly half of human primary lung tumors [49], are candidate PKCε effectors. Vav1 activation is regulated by tyrosine phosphorylation, and it may be also regulated by serine/threonine kinases [57]. Very recently, Vav1 was found to be associated with PKC θ, a member of the nPKC family closely related to PKCε [58]. Other small GTPase GEFs regulated by serine/threonines kinases include STEF, β1-Pix, and GEF-H1 [59], [60], [61]. Moreover, phorbol esters can activate Rho GTPases through mechanisms that involve Rho-GEF activation [62], [63]. Conceivably, similar mechanisms may take place on Rac-GEFs via specific phosphorylation by PKCε. PKCε may also modulate Rac-GAPs that control the rate of GTP hydrolysis from the GTPase. For example, phorbol esters stimulate the phosphorylation of the Rho-GAP DLC1, leading to its relocalization and suppression of GAP activity [64]. Very recently, our laboratory identified a PKC-mediated phosphorylation mechanism that modulates the association of the Rac-GAP β2-chimaerin to the plasma membrane [6]. The identification of those substrates of PKCε implicated in the regulation of the Rac signaling pathway will certainly provide important insights into the mechanisms of lung cancer metastasis.

Finally, our studies may have significant therapeutic implications. Several modulators of PKC activity have been clinically tested for different types of malignancies with variable degrees of success [32], [65], [66]. Despite the established oncogenic and invasive activity of PKCε, to our knowledge PKCε inhibitors have not been tested as anti-cancer agents. This may be largely due to the failure to develop selective inhibitors directed against the ATP-binding site of PKCε, a shortcoming also observed for several other members of the PKC family. Nonetheless, here we found that the specific PKCε translocator inhibitor εV1-2 impairs Rac1 activation and migration in a similar manner as observed upon delivery of PKCε RNAi duplexes. In preliminary studies we found that systemic delivery of εV1-2 fused to the permeable peptide TAT inhibits NSCLC xenograft growth in nude mice (M.C.C., C.L.H., and M.G.K., unpublished observations). Our studies may establish a framework for designing PKCε inhibitors as anti-cancer agents, particularly for those malignancies such as lung cancer for which there are currently limited therapeutic options available.

## Supporting Information

Figure S1 **PKC activation induces lamelipodia and ruffles in NSCLC cells.** Cells were serum starved for 24 h, pretreated with the pan-PKC inhibitor GF109203X (5 µM, 30 min) and stimulated with PMA (100 nM, 30 min) in the presence of the inhibitor. After washing, cells were fixed and stained with rhodamine-phalloidin. A representative micrograph is shown (n = 3).
(TIF)

Figure S2 **PKC activation accelerates wound closure in NSCLC cells.** Cells were serum starved for 24 h, pretreated with the pan-PKC inhibitor GF109203X (5 µM, 30 min) and stimulated with PMA (100 nM, 30 min). After washing, monolayers were scraped and the closure of the wound was followed for 9 h (A549) or 20 h (H358 and H1299). A representative micrograph is shown.
(TIF)

Figure S3 **Human NSCLC cell lines express high levels of PKCε compared to non-tumorigenic immortalized bronchioepithelial cells (HBE).** Expression was determined by Western blot. Similar results were observed in 3 independent experiments.
(TIF)

Figure S4 **PKCε mediates Rac activation and Rac-mediated responses induced by PMA in H322 cells.** H322 cells were transfected with either PKCε RNAi (ε1 or ε2) or control (*CTRL*) RNAi and serum starved for 24 h. A) Cells were treated with PMA (100 nM, 30 min), fixed, and stained with phalloidin-rhodamine. Representative micrographs are shown (n = 3). B) Quantification of H322 cells bearing ruffles, expressed as mean ± S.D. of 3 individual experiments. **, p<0.01; ***, p<0.001. C) Closure of wounds in response to PMA (100 nM, 30 min) was recorded at 20 h. Experiments were carried out in triplicate plates. A representative micrograph is shown (n = 3).
(TIF)

Figure S5 **Cell viability with treatments used for Boyden chamber experiments.** A) *Left panel*, A549 cells were serum starved for 24 h, trypsinized and plated for either MTT assays in the presence of the indicated inhibitors (16 h). Data are expressed as mean ± S.D. (n = 6). *Right panel*, PKCε depletion achieved by transient transfection of siRNA. B) Cells were seeded in Boyden chambers in the presence of vehicle or GF109203X (5 µM). FBS (10%) was added to the lower compartment. Migratory cells were determined 16 h later. *Left panel*, representative experiments. *Right panel*, quantification of migratory cells. Data are expressed as mean ± S.E.M. (n = 3). ***, p<0.001.
(TIF)

Figure S6 **PKCε overexpression enhances wound closure induced by serum in NSCLC cells.** Cells were infected with adenovirus (AdV) coding the indicated proteins, serum starved for 24 h and assayed for migration in response to serum (FBS) with either wound assays or Boyden chambers. Cells were infected with a control (LacZ) or PKC AdVs at multiplicities of infection = 100 pfu/cell. A) Expression of PKC isozymes at the day of the experiment. B) Representative micrographs for a wound assay in A549 infected with LacZAdV or PKCε AdV. C) Quantification of wound closure induced by 10% FBS for H358 or A549 cells infected with LacZ, PKCα, PKCδ or PKCε AdVs. Data are expressed as mean ± S.E.M. (n = 3). *, p<0.05; **, p<0.01; ***, p<0.001.
(TIF)

Figure S7 **εV1-2 concentration-response in migration assays.** H358 cells were seeded in Boyden chambers in the presence of TAT 10 µM or εV1-2 (0.1–10 µM). FBS (10%) was added to the lower compartment. Migratory cells were measured at 16 h. *Left panel*, representative experiments. *Right panel*, quantification of migratory cells. Data are expressed as mean ± S.E.M. (n = 3). ***, p<0.001.
(TIF)

Figure S8 **Detection of ECM-proteases secreted to conditioned media (CM) either from control or PKCε-depleted A549 cells.** A549 cells transfected with either PKCε RNAi or control duplexes, and CM was collected as described in Experimental Procedures. A) ECM-proteases were detected in CM from control (*CTRL-CM*) or PKCε-depleted cells (*PKCε-CM*) using a protein array detection system from RayBiotech. *IC*, internal positive control. B) MMP9 activity was assayed by zymography. Two additional experiments gave similar results.
(TIF)
