# Supplementary figures and images for: Non-Small Cell Lung Carcinoma Cell Motility, Rac Activation and Metastatic Dissemination Are Mediated by Protein Kinase C Epsilon
Source: PLoS One. 2012 Feb 27;7(2):e31714. doi: 10.1371/journal.pone.0031714 (PMC3288050; doi:10.1371/journal.pone.0031714)

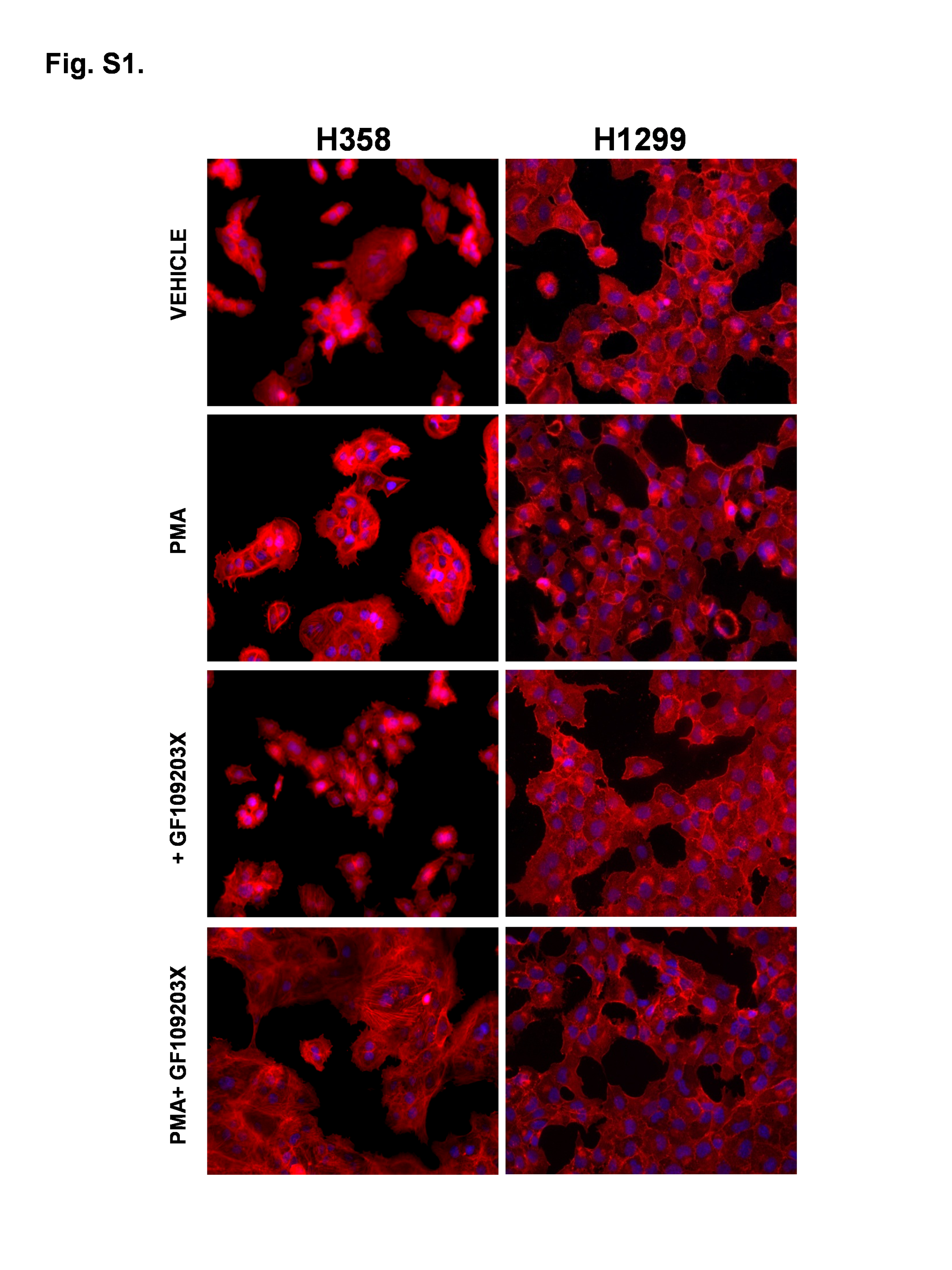

Supplement: Figure S1 — PKC activation induces lamelipodia and ruffles in NSCLC cells. Cells were serum starved for 24 h, pretreated with the pan-PKC inhibitor GF109203X (5 µM, 30 min) and stimulated with PMA (100 nM, 30 min) in the presence of the inhibitor. After washing, cells were fixed and stained with rhodamine-phalloidin. A representative micrograph is shown (n = 3). (TIF) [file pone.0031714.s001.tif]

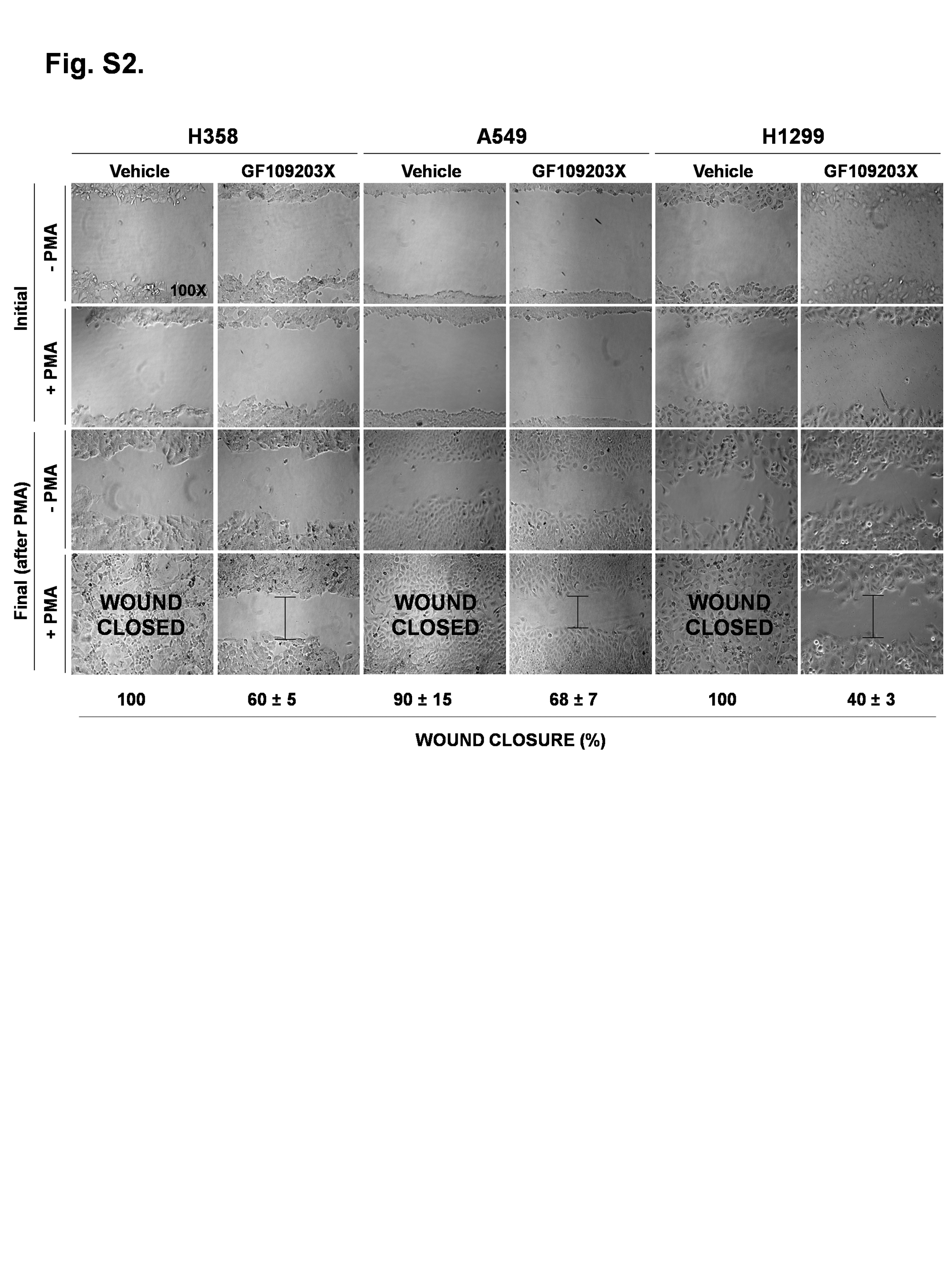

Supplement: Figure S2 — PKC activation accelerates wound closure in NSCLC cells. Cells were serum starved for 24 h, pretreated with the pan-PKC inhibitor GF109203X (5 µM, 30 min) and stimulated with PMA (100 nM, 30 min). After washing, monolayers were scraped and the closure of the wound was followed for 9 h (A549) or 20 h (H358 and H1299). A representative micrograph is shown. (TIF) [file pone.0031714.s002.tif]

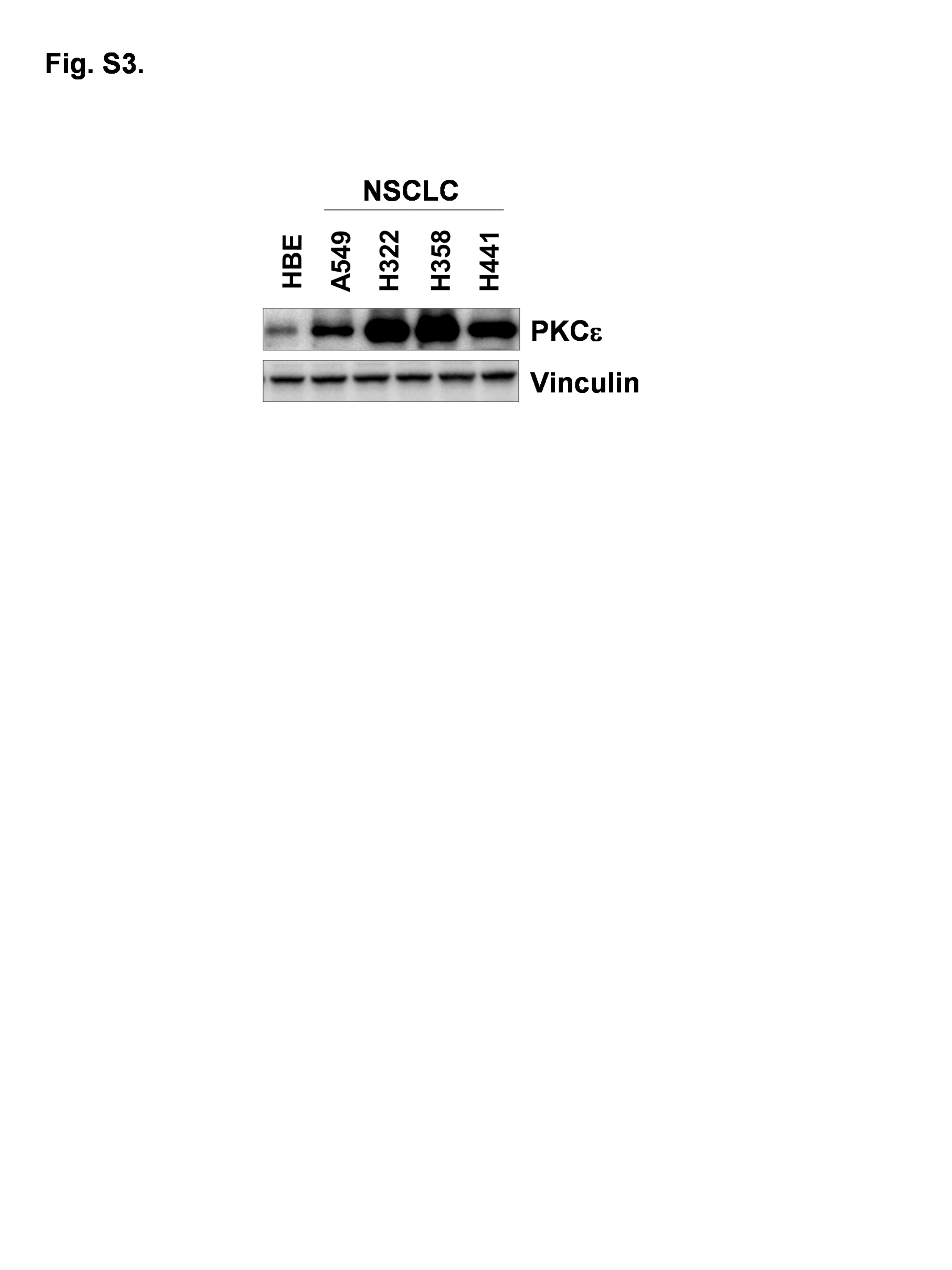

Supplement: Figure S3 — Human NSCLC cell lines express high levels of PKCε compared to non-tumorigenic immortalized bronchioepithelial cells (HBE). Expression was determined by Western blot. Similar results were observed in 3 independent experiments. (TIF) [file pone.0031714.s003.tif]

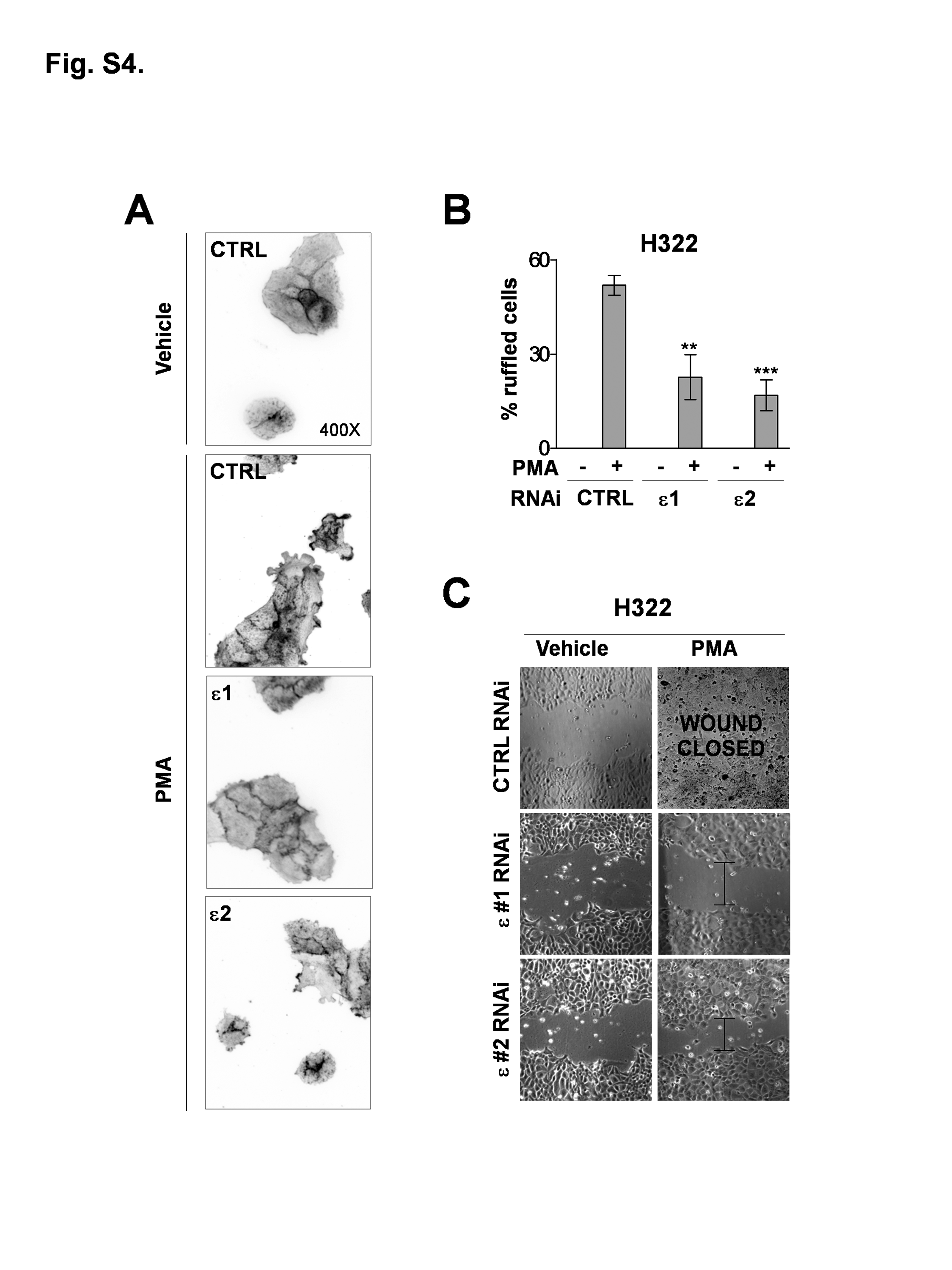

Supplement: Figure S4 — PKCε mediates Rac activation and Rac-mediated responses induced by PMA in H322 cells. H322 cells were transfected with either PKCε RNAi (ε1 or ε2) or control (CTRL) RNAi and serum starved for 24 h. A) Cells were treated with PMA (100 nM, 30 min), fixed, and stained with phalloidin-rhodamine. Representative micrographs are shown (n = 3). B) Quantification of H322 cells bearing ruffles, expressed as mean ± S.D. of 3 individual experiments. **, p<0.01; ***, p<0.001. C) Closure of wounds in response to PMA (100 nM, 30 min) was recorded at 20 h. Experiments were carried out in triplicate plates. A representative micrograph is shown (n = 3). (TIF) [file pone.0031714.s004.tif]

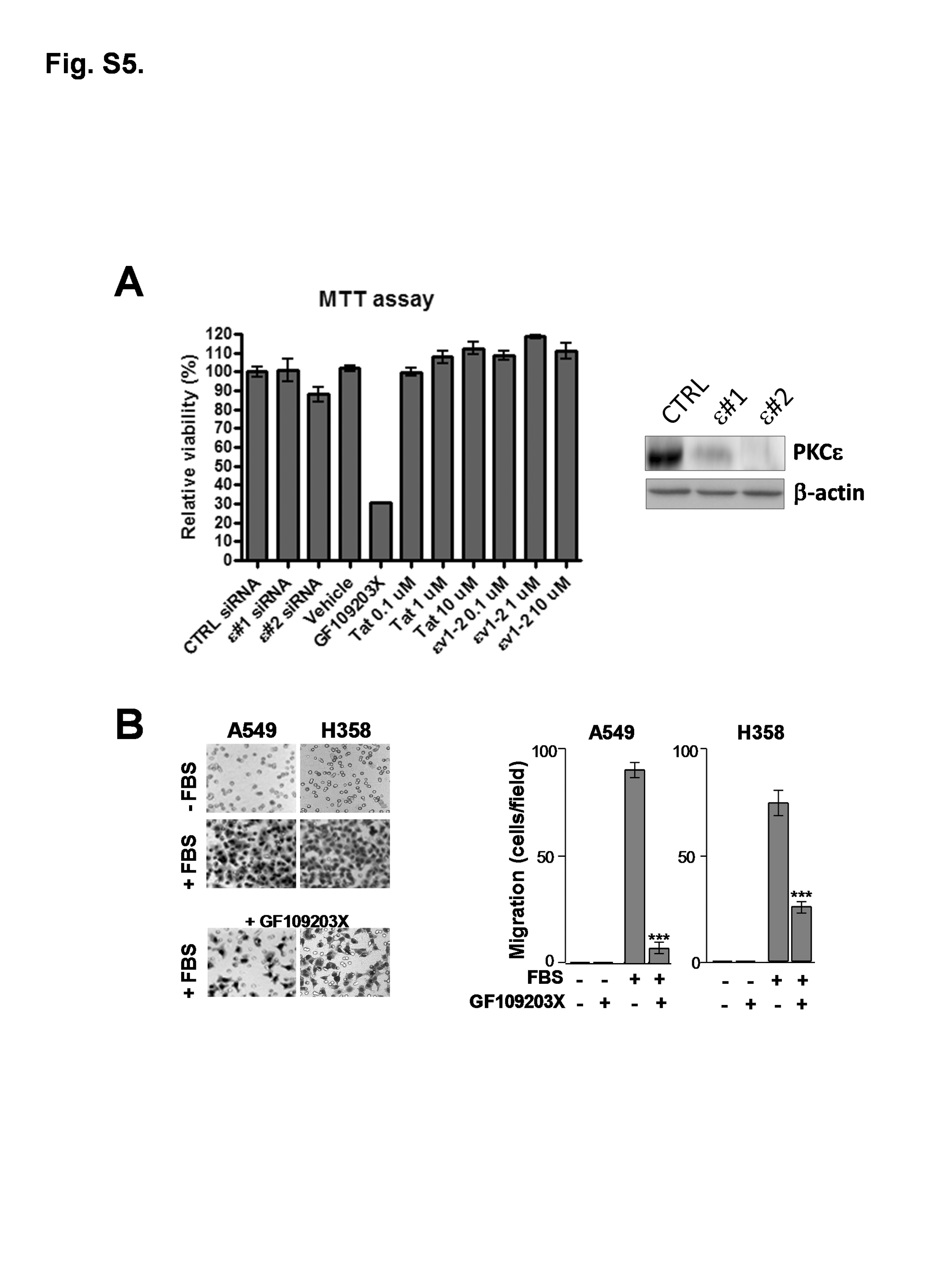

Supplement: Figure S5 — Cell viability with treatments used for Boyden chamber experiments. A) Left panel, A549 cells were serum starved for 24 h, trypsinized and plated for either MTT assays in the presence of the indicated inhibitors (16 h). Data are expressed as mean ± S.D. (n = 6). Right panel, PKCε depletion achieved by transient transfection of siRNA. B) Cells were seeded in Boyden chambers in the presence of vehicle or GF109203X (5 µM). FBS (10%) was added to the lower compartment. Migratory cells were determined 16 h later. Left panel, representative experiments. Right panel, quantification of migratory cells. Data are expressed as mean ± S.E.M. (n = 3). ***, p<0.001. (TIF) [file pone.0031714.s005.tif]

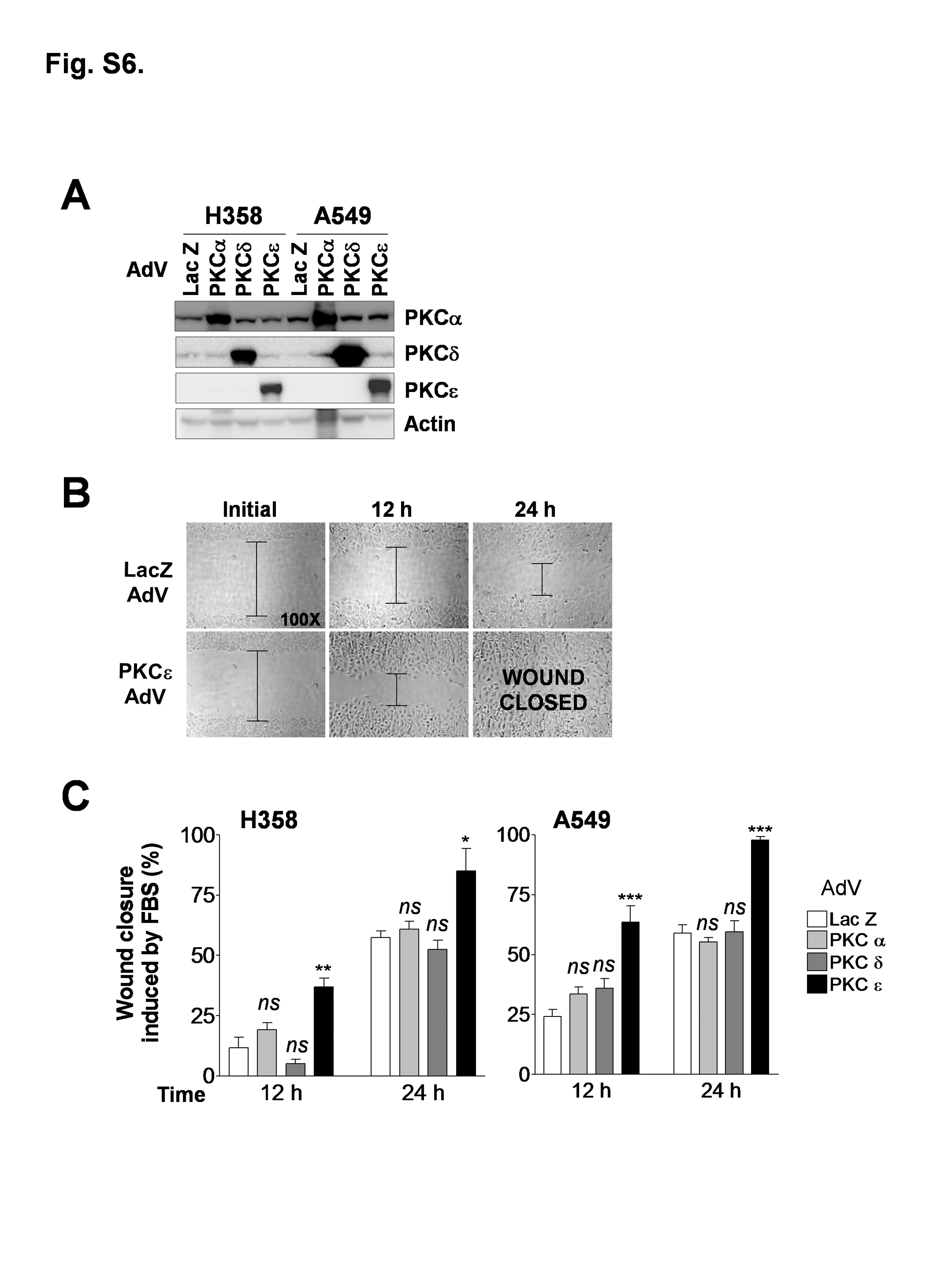

Supplement: Figure S6 — PKCε overexpression enhances wound closure induced by serum in NSCLC cells. Cells were infected with adenovirus (AdV) coding the indicated proteins, serum starved for 24 h and assayed for migration in response to serum (FBS) with either wound assays or Boyden chambers. Cells were infected with a control (LacZ) or PKC AdVs at multiplicities of infection = 100 pfu/cell. A) Expression of PKC isozymes at the day of the experiment. B) Representative micrographs for a wound assay in A549 infected with LacZAdV or PKCε AdV. C) Quantification of wound closure induced by 10% FBS for H358 or A549 cells infected with LacZ, PKCα, PKCδ or PKCε AdVs. Data are expressed as mean ± S.E.M. (n = 3). *, p<0.05; **, p<0.01; ***, p<0.001. (TIF) [file pone.0031714.s006.tif]

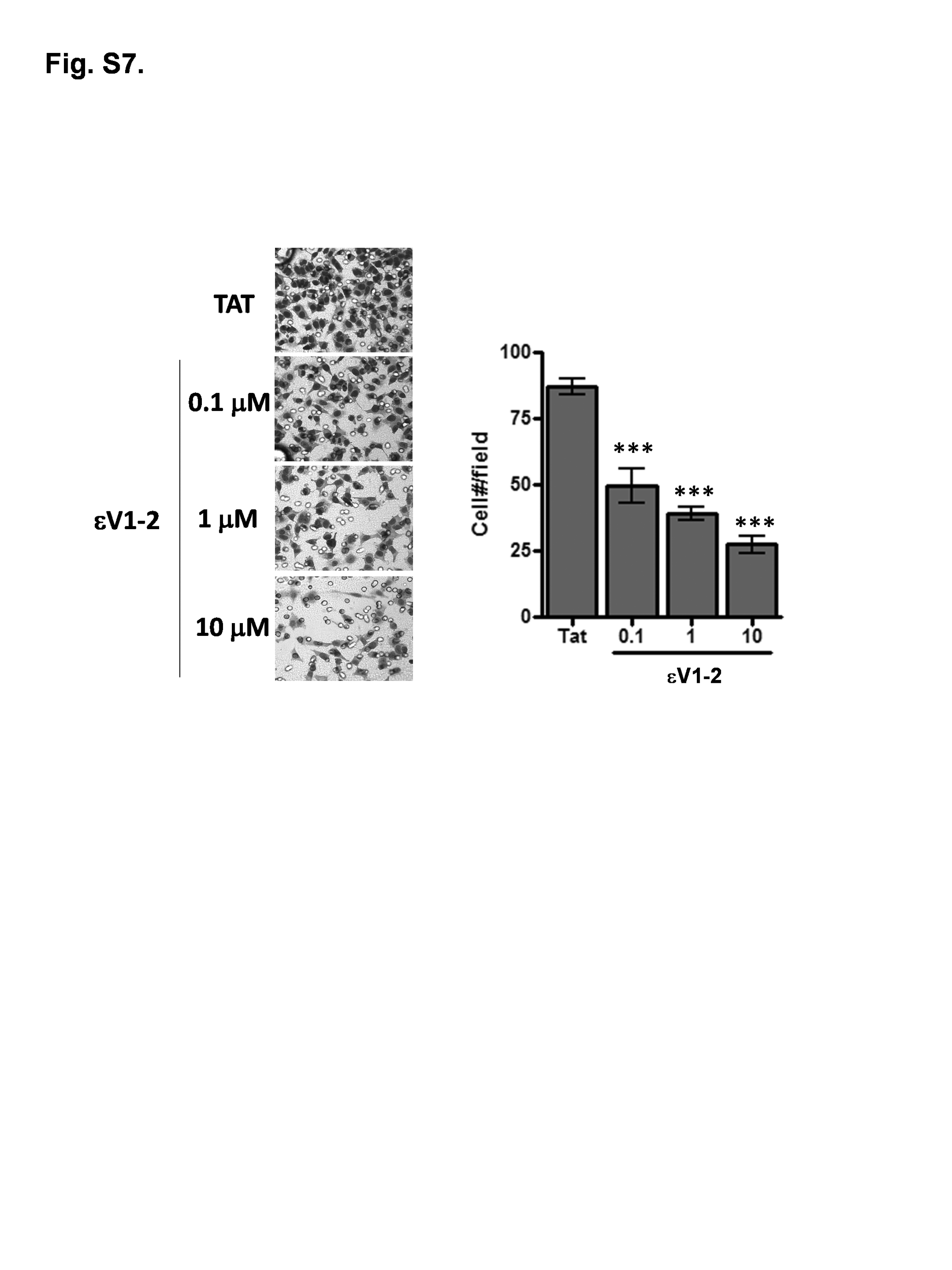

Supplement: Figure S7 — εV1-2 concentration-response in migration assays. H358 cells were seeded in Boyden chambers in the presence of TAT 10 µM or εV1-2 (0.1–10 µM). FBS (10%) was added to the lower compartment. Migratory cells were measured at 16 h. Left panel, representative experiments. Right panel, quantification of migratory cells. Data are expressed as mean ± S.E.M. (n = 3). ***, p<0.001. (TIF) [file pone.0031714.s007.tif]

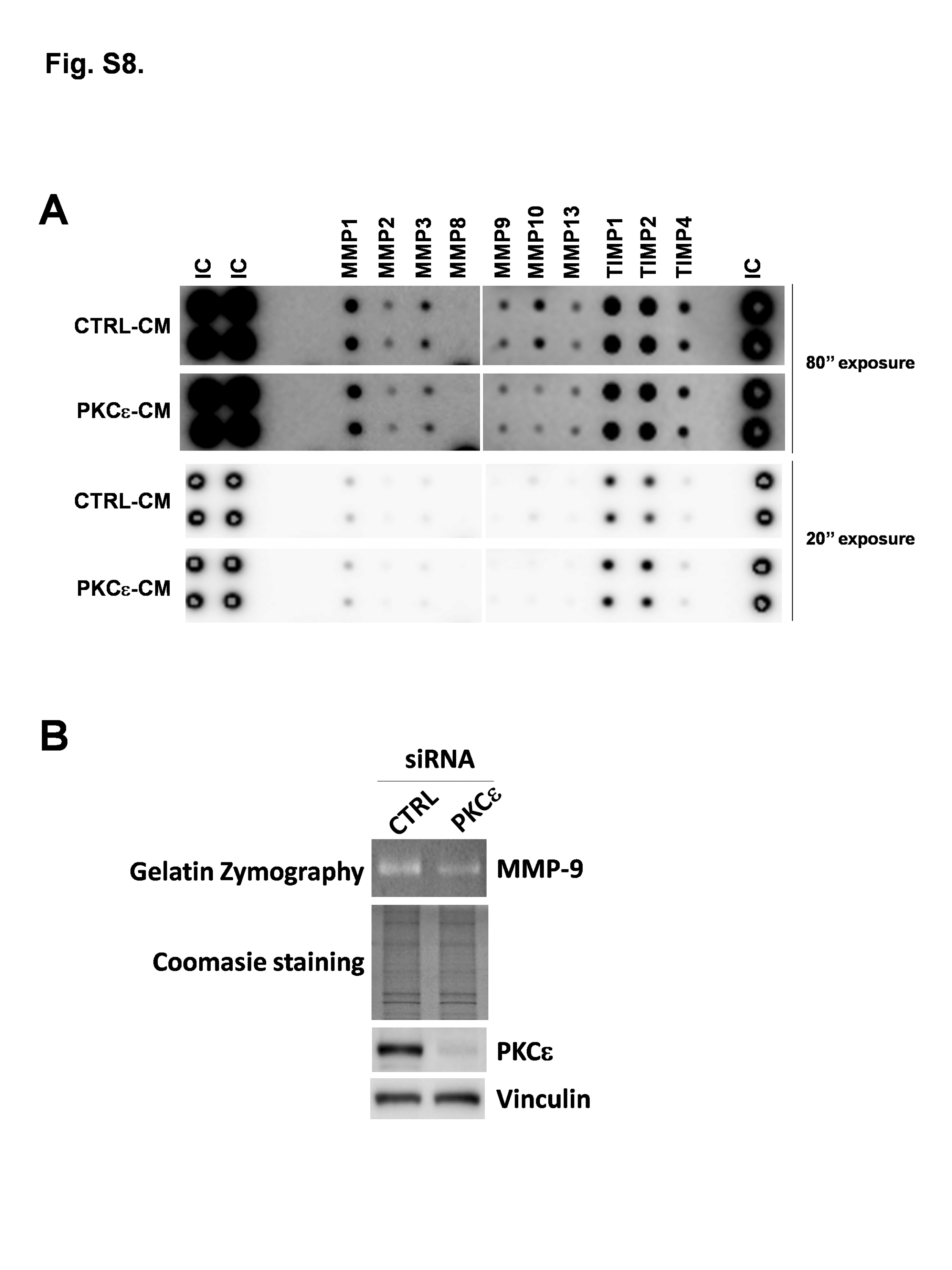

Supplement: Figure S8 — Detection of ECM-proteases secreted to conditioned media (CM) either from control or PKCε-depleted A549 cells. A549 cells transfected with either PKCε RNAi or control duplexes, and CM was collected as described in Experimental Procedures. A) ECM-proteases were detected in CM from control (CTRL-CM) or PKCε-depleted cells (PKCε-CM) using a protein array detection system from RayBiotech. IC, internal positive control. B) MMP9 activity was assayed by zymography. Two additional experiments gave similar results. (TIF) [file pone.0031714.s008.tif]
